# FG-4592 relieves diabetic kidney disease severity by influencing metabolic profiles via gut microbiota reconstruction in both human and mouse models

**DOI:** 10.3389/fphys.2023.1195441

**Published:** 2023-08-15

**Authors:** Yumin Jiang, Wen Cui, Yiding Zhang, Ting Wang, Xuejun Zheng, Huangmin Li, Jin Shang

**Affiliations:** ^1^ Department of Emergency Intensive Care Unit, The First Affiliated Hospital of Zhengzhou University, Zhengzhou, Henan, China; ^2^ Department of Nephrology, The First Affiliated Hospital of Zhengzhou University, Zhengzhou, Henan, China; ^3^ Zhengzhou University, Zhengzhou, Henan, China

**Keywords:** diabetic kidney disease, FG-4592, gut microbiota, untargeted metabolomics analysis, mechanism

## Abstract

**Objective:** Diabetic kidney disease (DKD) is one of the most prevalent complications of diabetes mellitus (DM) and is highly associated with devastating outcomes. Hypoxia-inducible factor (HIF), the main transcription factor that regulates cellular responses to hypoxia, plays an important role in regulating erythropoietin (EPO) synthesis. FG-4592 is the HIF stabilizer that is widely used in patients with renal anemia. We investigated the effect of FG-4592 on DKD phenotypes and the pharmacologic mechanism from the perspective of gut microbiota and systemic metabolism.

**Design:** We collected the clinical data of 73 participants, including 40 DKD patients with combined renal anemia treated with FG-4592, and 33 clinical index-matched DKD patients without FG-4592 treatment from The First Affiliated Hospital of Zhengzhou University at the beginning and after a 3–6-month follow-up period. We established DKD mouse models treated by FG-4592 and performed fecal microbiota transplantation from FG-4592-treated DKD mice to investigate the effects of FG-4592 on DKD and to understand this mechanism from a microbial perspective. Untargeted metabolome–microbiome combined analysis was implemented to globally delineate the mechanism of FG-4592 from both microbial and metabolomic aspects.

**Result:** DKD phenotypes significantly improved after 3–6 months of FG-4592 treatment in DKD patients combined with renal anemia, including a decreased level of systolic blood pressure, serum creatinine, and increased estimated glomerular infiltration rate. Such effects were also achieved in the DKD mouse model treated with FG-4592 and can be also induced by FG-4592-influenced gut microbiota. Untargeted plasma metabolomics-gut microbiota analysis showed that FG-4592 dramatically altered both the microbial and metabolic profiles of DKD mice and relieved DKD phenotypes via upregulating beneficial gut microbiota-associated metabolites.

**Conclusion:** FG-4592 can globally relieve the symptoms of DKD patients combined with renal anemia. In the animal experiment, FG-4592 can reconstruct the intestinal microbial profiles of DKD to further upregulate the production of gut-associated beneficial metabolites, subsequently improving DKD phenotypes.

## 1 Background

Diabetic kidney disease (DKD)—also known as diabetic nephropathy (DN)—remains the leading worldwide cause of chronic kidney disease (CKD) and end-stage renal disease (ESRD). DKD usually occurs in patients with diabetes mellitus (DM) whose blood glucose is not adequately controlled and is one of the major causes of death in DM patients (Gross et al., 2005). As a clinical syndrome, DKD consists of persistent proteinuria, sustained reduction in GFR, increased blood pressure, and prevalent cardiovascular events and their related mortality (Lin et al., 2018). Considering the devastating outcomes and poor prognosis of DKD, it is urgent to decipher its accurate mechanism.

FG-4592 is a prolyl hydroxylase (PHD) inhibitor that stabilizes the level of hypoxia-inducible factor (HIF), the transcription factor that drives the expression of genes contributing to erythropoiesis (Akizawa et al., 2019; Chang et al., 2019). Given that HIF is responsible for regulating a broad range of downstream effect genes to adapt hypoxia, HIF stabilization induced by FG-4592 can result in a series of off-target effects while it treats anemia. According to previous studies, FG-4592 can reduce renovascular resistance and increase glomerular filtration by stimulating nitric oxide generation (Burmakin et al., 2021). Furthermore, FG-4592 significantly reduces the expression of fibronectin and TGF-β1 in the serum of CKD patients with anemia, which in turn improves renal fibrosis (Zheng et al., 2022). Considering that renal anemia is a major complication in CKD, while DKD remains the leading cause of CKD (Tsai and Tarng, 2019), applying FG-4592 in anemic DKD patients may be beneficial to not only improve anemia but also to relieve other clinical phenotypes of DKD.

Gut microbiota, the crucial commensal microbial community located in the human gastrointestinal tract, have been reported to be sensitive and adaptative to altered intestinal oxygen states and have presented a reconstructed microbial structure with a higher abundance of Proteobacteria and Actinobacteria in hyperoxic animals *versus* a higher abundance of Firmicutes in hypoxic mice (He et al., 1999; Moreno-Indias et al., 2015). Kelly et al. (2015) reported that germ free mice and antibiotic-treated mice showed relatively reduced HIF-1 activation. Moreover, the stabilization of HIF1 induced by butyrate—a gut microbiota-derived short-chain fatty acid (SCFA)—shifts the metabolic state of intestinal epithelial cells into anaerobic glycolysis, impacting both microorganisms and host cells (Kelly et al., 2015; Rivera-Chávez et al., 2016; Byndloss et al., 2017; Reese et al., 2018; Cevallos et al., 2021). Therefore, the effects of HIF stabilizers on gut microbiota and systemic plasma metabolic profiles in DKD have strongly attracted our attention.

In this study, we enrolled DKD patients with renal anemia who received (n = 40) or did not receive (n = 33) FG-4592; we collected the clinical data at baseline and after 3–6 months follow-up to clarify the influences of FG-4592 on DKD-relevant signatures. We then constructed a DKD mouse model to repeat the evaluation and confirm the impact of FG-4592 on DKD phenotypes. Fecal and plasma samples of the mice were collected and subjected to gut microbiota-plasma untargeted metabolomic combining analysis. This study aimed to comprehensively delineate intestinal microbial community features and systemic plasma metabolic profiles and to uncover the potential mechanistic underpinnings of FG-4592’s influence on DKD phenotypes specifically from the microbial and metabolic perspectives.

## 2 Materials and methods

### 2.1 Participant enrollment

Patients with combined DKD and renal anemia who were hospitalized in The First Affiliated Hospital of Zhengzhou University from April 2020 to October 2022 were enrolled. Our study followed the Helsinki Declaration. The Ethics Review Committee approved all experimental processes (2019-KY-361). All participants signed a written informed consent.

The diagnostic criteria for DKD were at least a 5-year history of diabetes complicated with repeated albuminuria (urinary protein/creatinine≥30 mg/g) or macro-protein urine (Liu et al., 2018; Tao et al., 2019). Diagnostic criteria for renal anemia were Hb < 120 g/L for women or Hb < 130 g/L for men with estimated glomerular filtration rate (eGFR) < 90 mL/min/1.75 m^2^ or serum creatinine >300 μmol/L/L. The exclusion criteria of the study were: 1) patients combined with other secondary kidney diseases (e.g., infection, lupus, vasculitis, and hepatitis B) or other causes of anemia (e.g., nutritional anemia, acute/chronic blood loss, and autoimmune anemia); 2) pure primary glomerulonephritis confirmed by renal biopsy; 3) patients whose DKD was controlled and whose urine protein turned negative when enrolled; and 4) incomplete information. Finally, we prospectively recruited 112 DKD patients with renal anemia. The clinical baseline characteristics were collected with the fecal samples and included gender, age, systolic blood pressure (SBP), diastolic pressure (DBP), hemoglobin (Hb), glycosylated hemoglobin (Ghb), serum creatinine (Cr), estimated glomerular filtration rate (eGFR), albumin (Alb), and 24 h urine protein (24hpro).

### 2.2 Animal modeling and clinical evaluation

All animal experimentation was approved by the Ethical Committee of Experimental Animal Care of The First Affiliated Hospital of Zhengzhou University (2021-KY-0162). We purchased 32 6-week-old male C57/BL6 mice from the Animal Center of Zhengzhou University. Broad-spectrum antibiotics gavage was used to clear gut microbiota and establish germ free (GF) mice, as described by Miyauchi et al. (2020) and Peng et al. (2021). Fecal microbiota transplantation (FMT) and gut microbiota clearance were implemented on the mice to investigate the connection between FG-4592, intestinal microbiota, and DKD phenotypes. Intraperitoneal streptozotocin (STZ, from Sigma-Aldrich) injections combined with high-fat diet (HFD) feeding (45% fat, XTHF45-1, Jiangsu, China) were used to induce DKD. The injection dose of STZ was 55 mg/1000 g body weight, lasting for 7 days. Blood glucose measurements were conducted after injections ceased for 7 consecutive days to determine whether the model was successfully established. On the last STZ injection, FG-4592 was intragastrically administered daily (50 mg/kg/day) as described by Kabei et al. (2020) for 14 days. The antimicrobial solution comprised ampicillin 0.1 g/L, vancomycin 0.5 g/L, neomycin 1 g/L, and metronidazole 1 g/L (obtained from Sigma-Aldrich), replacing daily purified water intake. The control group was fed a chow diet (SWS9102, Jiangsu, China) and intragastrically administered a saline vehicle. The fecal samples of mice were collected and subjected to 16S rRNA gene sequencing. Detailed information of the modeling process, fecal bacteria solution preparation, antibiotic formula, and sample collection are shown in Supplementary File S1. Random blood glucose levels and body weight were measured weekly. We collected spot urines and total urinary protein/urinary creatinine (T/Cr) to evaluate the severity of proteinuria in DKD and presented these as scatter charts. We used analysis of variances (ANOVA) to test the difference in body weight, blood glucose, and T/Cr in the three groups.

FG-4592 (Evrenzo^®^, AstraZeneca, and FibroGen) was used in our animal experiment. The mice were randomly divided into four groups according to the gavage method: 1) healthy control (Con) (n = 10); 2) DKD (n = 10); 3) DKD treated with FG-4592 (DKD-FG) (n = 8); 4) DKD mice whose gut microbiota were cleared by broad-spectrum antibiotics and then transplanted with the gut microbiota of Group 3 (DKD-FG-FMT) (n = 4). Detailed information about the experiment design and sample collection plan is given in Supplementary File S1.

### 2.3 16S rRNA gene sequencing and OTU clustering

A total of 23 mouse fecal samples (seven from the Con group, eight from the DKD group, and eight from the DKD-FG group) were collected and subjected to 16S rRNA gene sequencing. DNA extraction from fecal samples was performed as described by Chen et al. (2011) and Peng et al. (2018) using an E.Z.N.A.^®^ Stool DNA Kit (Omega Bio-tek, Inc., GA). The detailed process of DNA extraction is illustrated in Supplementary Document 2. Shanghai MoBio Biomedical Technology Co. Ltd. provided technical support using the Miseq platform (Illumina Inc., United States) per the manufacturer’s protocols. F1 and R2 primers (5′- CCTACGGGNGGCWGCAG -3′ and 5′-GACTACHVGGGTATCTAATCC-3′), which correspond to positions 341 to 805 in the *Escherichia coli* 16S rRNA gene, were used to amplify the V3–V4 region by PCR. PCR amplification of this region of the 16S rRNA gene and Illumina paired-end sequencing were performed according to a previous description. To obtain clean data, we treated the raw data using USEARCH (version 11.0.667) thus: 1) sequences of each sample were extracted using each index with zero mismatch; 2) sequences with overlap less than 16 bp were discarded; 3) sequences less than 400 bp after merge were discarded; 4) sequences with an overlap error rate greater than 0.1 were discarded. The quality-filtered sequences were clustered into unique sequences and sorted by decreasing abundance. According to the UPARSE OTU analysis pipeline, the representative sequences were identified using UPARSE, and singletons were omitted in this step. Operational taxonomic units (OTUs) were obtained based on 97% similarity after chimeric sequences were removed using UPARSE (version 7.1 http://drive5.com/uparse/) and were annotated using the SILVA reference database (SSU138) (Edgar, 2013; Sung et al., 2017). The phylogenetic affiliation of the 16S rRNA gene sequence was analyzed with a confidence threshold of 70% (Wang et al., 2007). To analyze the phylogenetic affiliation of the 16S rRNA gene sequence, we used RDP Classifier (http://rdp.cme.msu.edu/) against the SILVA (SSU123) 16S rRNA database with a confidence threshold of 70%. Gut microbiota composition and functional changes were compared between different groups. The non-parametric Mann–Whitney *U* test (R 3.6.0 package stats) was used to test for significant differences between two groups. Multiple groups were compared using a nonparametric Kruskal–Wallis test. Both Bray–Curtis, weighted, and unweighted UniFrac dissimilarities were calculated in QIIME (v1.9.1). Principal coordinate analysis (PCoA) plots and permutational multivariate analysis of variance (PERMANOVA) used to test for statistical significance between the groups using 10,000 permutations were generated in the R (version 3.6.0) package vegan 2.5–7. Linear discriminant analysis (LDA) effect size (LEfSe) was used to detect taxa with differential abundance among groups (lefse 1.1, https://github.com/SegataLab/lefse). PICRUSt2 v2.4.1 (https://github.com/picrust/picrust2/wiki) was used to predict functional abundances based on 16S rRNA gene sequences. All codes of R packages were uploaded to GitHub (https://github.com/Neal050617/16S-rDNA-analysis).

### 2.4 Microbial community analysis

Bacterial richness and diversity were estimated separately by Chao/observed OTU and Shannon/Simpson indices in our study. The Mann–Whitney *U* test was used to compare the OTU differences between the two groups. Differences between the three groups were compared using the Kruskal–Wallis test. Bray–Curtis dissimilarity and (un)weighted UniFrac distances were calculated in QIIME to assess beta diversity. In addition, principal coordinate analysis (PCoA) and non-metric multidimensional scaling analysis (NMDS) were performed to aid the interpretation of bacterial distribution among the three groups. Correspondingly, we used PERMANOVA and analysis of similarities (ANOSIM) to evaluate the statistical significance of the differences between the three groups. Phylogenetic investigation of communities by reconstruction of unobserved states (PICRUSt) was used to predict markedly enriched KEGG metabolic pathways (LDA scores>2.0 and *p*-value<0.05). Detailed statistical bioinformatic analysis and tests are described in Supplementary File S2.

### 2.5 Metabolic profile delineation of mice models

The mouse 17 plasma samples (five from the Con group, six from the DKD group, and six from the DKD-FG group) were subjected to ultra-high-performance liquid chromatography-mass spectrometry (UPLC-MS, Thermo, Q Exactive)-based untargeted metabolomic analysis to globally describe the plasma’s metabolic features. All detected metabolites were identified by MS and MS/MS fragments through Progenesis QI (WaterCorporation, Milford, United States) with several mainstream public databases (http://www.hmdb.ca/, https://metlin.scripps.edu/). Principal component analysis (PCA) and orthogonal partial least-squares discrimination analysis (OPLS-DA) were used to identify the disparity of plasma metabolites. Based on OPLS-DA analysis, metabolites with variable importance in projection (VIP) > 1 were recognized as important variables. VIP represents the ability to extract variables of differentiation among groups. Important differential metabolites were defined as those with VIP>1.0 obtained from OPLS-DA and adjusted *p*-values < 0.05. Detailed information of chemicals and equipment, sample processing, UPLC-MS analysis, and bioinformatic and statistical analysis are in Supplementary File S3.

### 2.6 Statistics

Data collected by biochemical assay were expressed as mean ± SEM. Statistical analyses were performed using SPSS 23.0 software. Comparisons between groups were measured by ANOVA. *p*-values <0.05 were considered statistically significant.

## 3 Results

### 3.1 FG-4592 can effectively improve the severity of DKD combined with renal anemia

We retrospectively recruited 73 DKD patients with renal anemia. Then, they were allocated to the FG-4592 (n = 40) or control groups (epo or no treatment, n = 33) according to their treatment strategies. As shown in Table 1, the clinical data of both the FG-4592 and Con groups completely matched at baseline. After 3–6 months follow-up (Table 2), the FG-4592 group showed significantly lower SBP and serum Cr and a significantly higher level of eGFR, indicating that FG-4592 can relieve severity in DKD patients with renal anemia compared with those patients who did not receive FG-4592. It should be mentioned that the FG-4592 group also presented relatively higher Hb and Alb and relatively lower DBP, Ghb, and 24 h-pro, even though the differences are not significant; this may be due to the relatively smaller size of the cohort. Considering that all clinical variables were comparable between the two groups, we reasonably concluded that FG-4592 treatment can comprehensively relieve severity for DKD patients with renal anemia. Furthermore, 73 DKD patients were also divided into male (n = 42) and female (n = 31) groups, which were used to evaluate the effects induced by FG-4592 in different sexes. Surprisingly, the aforementioned effects were only seen in the male group, which presented significantly lower SBP, DBP, and serum Cr, and higher serum Hb and eGFR after 3–6 months of FG-4592 treatment (Supplementary Figure S21). However, there were no significant differences in the clinical indices of the female group (Supplementary Figure S20).

**TABLE 1:** Demographic characteristics of DKD patients combined with renal anemia treated with FG-4592 group and control group at beginning.

Clinical index	FG-4592 group (n = 40)	Control group (n = 33)	*p*-value
Gender			0.639
Male	24	18	
Female	16	15	
Age	53.15 ± 9.05	54.55 ± 12.07	0.572
Height (cm)	168.00 (164.00–174.00)	167.00 (167.00–168.50)	0.303
Body weight (kg)	72.00 (62.00–78.00)	72.00 (72.00–73.75)	0.44
SBP (mmHg)	138.00 (125.00–150.00)	140.00 (132.00–156.50)	0.372
DBP (mmHg)	81.00 (78.00–88.00)	80.00 (72.00–91.50)	0.45
Hb (g/L)	91.00 (85.00–99.00)	91.00 (86.50–101.00)	0.637
Ghb (%)	6.20 (5.80–6.70)	6.29 (5.83–7.35)	0.583
Cr (μmol/L)	244.00 (156.00–384.00)	265.00 (182.50–338.50)	0.812
eGFR (mL/min/l.73m^2^)	19.700 (14.000–42.000)	17.609 (13.950–33.035)	0.698
Alb (g/L)	33.32 ± 7.00	30.84 ± 5.93	0.085
24hpro g)	4.78 (1.61–7.80)	4.44 (3.28–7.24)	0.65

Normal distribution was measured by the K-S test. Subsequent analysis between groups was completed by the LSD-t test. Variances between FG-4592 and control groups were analyzed by the *t*-test. χ^*2*^ test was used to compare categorical variables. SBP, systolic blood pressure; DBP, diastolic blood pressure; Hb, hemoglobin; Ghb, glycosylated hemoglobin; Cr, serum creatinine; eGFR, estimated glomerular filtration rate; Alb, serum albumin; 24 h-pro, 24 h-urine protein.

**TABLE 2:** Demographic characteristics of DKD patients combined with renal anemia treated with FG-4592 and control groups after 3–6 months follow-up.

Clinical index	FG-4592 group (n = 40)	Control group (n = 33)	*p*-value
Gender			0.639
Male	24	18	
Female	16	15	
Age	53.45 ± 8.79	54.70 ± 12.11	0.612
Height (cm)	168.00 (164.00–174.00)	167.00 (167.00–168.50)	0.303
Body weight (kg)	72.00 (62.00–78.00)	72.00 (72.00–73.75)	0.44
SBP (mmHg)	138.50 (128.50–144.75)	141.00 (131.50–162.50)	0.024
DBP (mmHg)	80.75 ± 8.99	84.70 ± 11.11	0.098
Hb (g/L)	99.50 (86.25–107.75)	92.00 (83.50–102.50)	0.178
Ghb (%)	6.200 (5.65–6.88)	6.58 (5.70–7.25)	0.235
Cr (μmol/L)	242.00 (163.50–375.75)	336.00 (228.90–512.50)	0.03
eGFR (mL/min/1.73m^2^)	24.000 (13.025–36.000)	14.281 (9.671–22.516)	0.013
Alb (g/L)	32.02 ± 6.42	31.36 ± 6.43	0.662
24hpro g)	3.60 (1.21–7.71)	5.30 (3.73–7.36)	0.126

Normal distribution was measured by the K-S test. Subsequent analysis between groups was completed by the LSD-t test. Variances between FG-4592 and control groups were analyzed by the *t*-test. χ^2^ test was used to compare categorical variables. SBP, systolic blood pressure; DBP, diastolic blood pressure; Hb, hemoglobin; Ghb, glycosylated hemoglobin; Cr, serum creatinine; eGFR, estimated glomerular filtration rate; Alb, serum albumin; 24 h-pro, 24 h-urine protein.

### 3.2 FG-4592 can improve DKD phenotypes via gut microbiota in DKD mice

**FIGURE 1:**
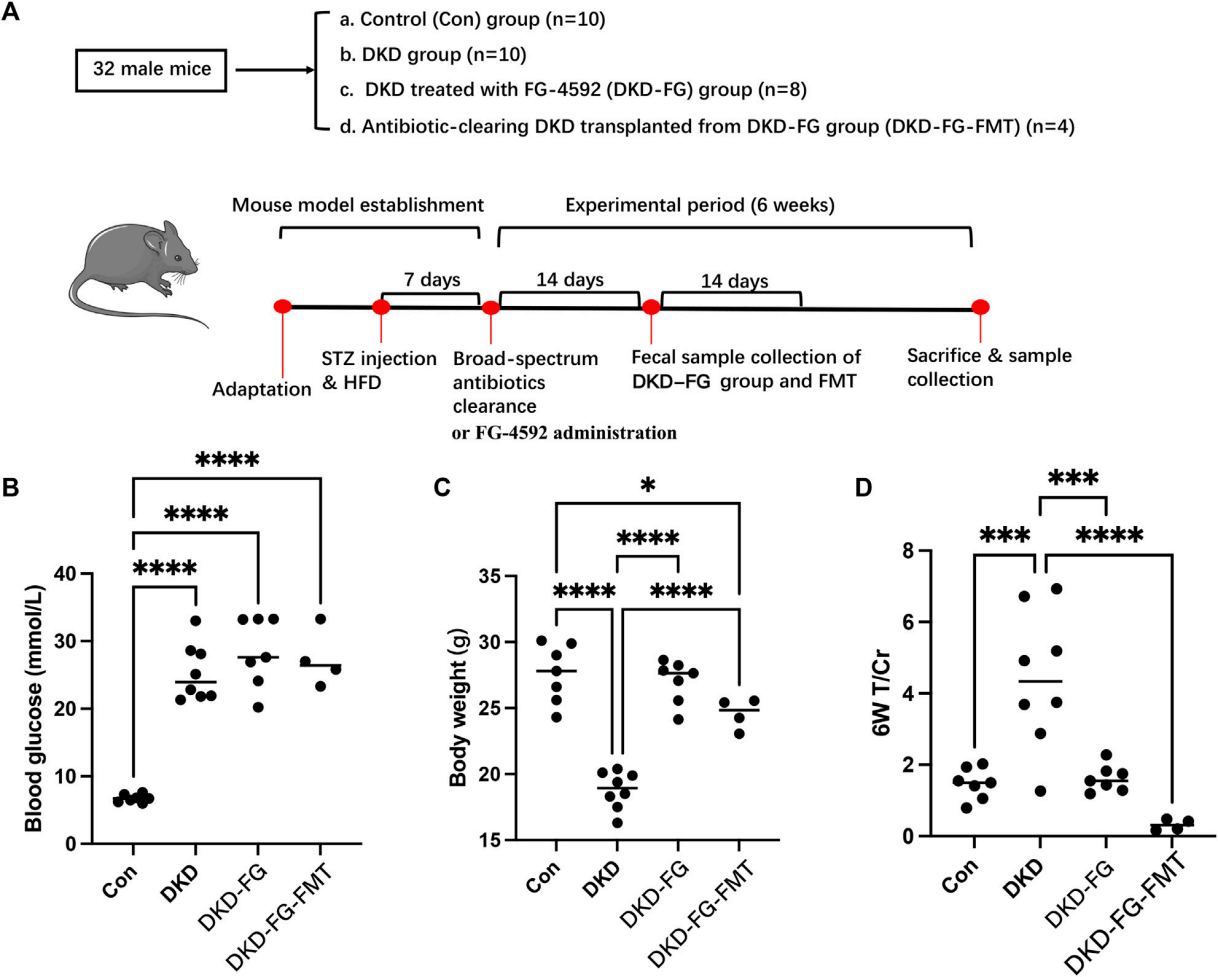
Experimental design and grouping process of animal experiment clinical phenotyping of DKD mouse models. Flow chart of the animal experiment **(A)**. Scatter plots show blood glucose **(B)**, body weight **(C)**, and urine total protein to creatinine ratio (T/Cr) **(D)** at the end of the experiment (6 weeks). DKD, diabetic kidney disease; GF, germ free; STZ, streptozotocin; FMT, fecal microbiota transplantation; T/Cr, total urinary protein/urinary creatinine. *p < 0.05; **p < 0.01; ***p < 0.001; ****p < 0.0001. ns, no significance.

According to the clinical indices collected from the DKD patients with renal anemia, FG-4592 treatment can globally relieve the phenotypes of DKD combined with renal anemia compared with the control group. To further investigate the mechanism of the DKD-relieving effect induced by FG-4592 and its potential relationship with gut microbiota, we established a mice model and implemented subsequent animal experiments. The grouping and model construction process are shown in Figure 1A. The experimental indices (including body weight, blood glucose, and T/Cr) of each group at the end of the experiment (6 weeks) are displayed in Figures 1B–D.

The blood glucose level of the DKD modeling groups was significantly increased over that of the Con group (Figure 1B). There was no significant difference among the DKD, DKD-FG, and DKD-FG-FMT groups, indicating that FG-4592 has no effect on the blood glucose level. Body weight was significantly higher in the DKD-FG-4592 group than in the DKD group and was comparable to the Con group. Surprisingly, FMT from the DKD-FG group can also effectively increase the body weight of DKD mice (Figure 1C), which was comparable with the DKD-FG group; this indicates that the body weight-improving effect of FG-4592 may rely on gut microbiota. A similar improving effect of FG-4592 also occurred in proteinuria. Successfully constructing a DKD model can result in proteinuria compared with the Con group, while FG-4592 treatment and FMT from the DKD-FG group can significantly reduce the level of T/Cr—comparable with the Con group (Figure 1D). Therefore, FG-4592 can dramatically relieve severity for DKD mice, which is consistent with the findings in our human cohort. Furthermore, such effects can be achieved by gut microbiota influenced by FG-4592.

### 3.3 FG-4592 reconstructs the gut microbiota structure of DKD mice

**FIGURE 2:**
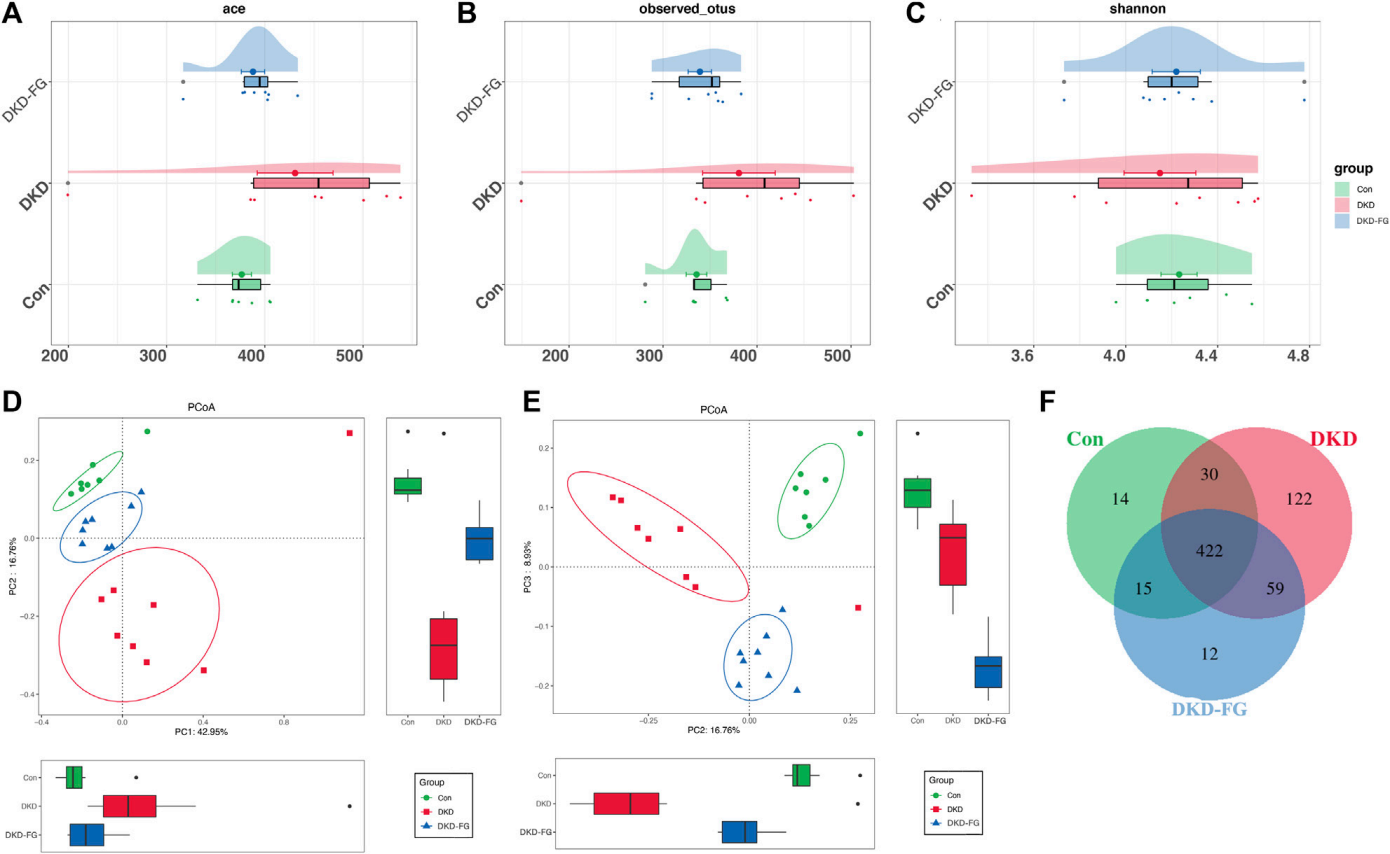
Bacterial diversity among Con (n = 7), DKD (n = 8), and DKD-FG-4592 groups (n = 8). α diversity: bacterial richness and diversity were evaluated by observed OTUs **(B)** and Shannon/Ace indices **(A, C)**, respectively. Venn diagram **(F)** showed observed OTUs among the three groups. β diversity: PCoA **(D, E)** analysis was measured by unweighted UniFrac distance at the OTU level. Adonis revealed that unweighted analysis taking OTU abundance into account could better reflect the spatial differences among the three groups (R2 = 0.274, *p* < 0.001). PCoA, principal coordinate analysis; PC, principal component, PC1, PC2, and PC3; Adonis, permutational/non-parametric multivariate analysis of variance.

After discovering that the DKD phenotype-relieving effects can only be achieved by the FG-4592-influenced gut microbiota, the 23 mouse fecal samples (seven from the Con group, eight from the DKD group, and eight from the DKD-FG group) were collected and subjected to 16S rRNA gene sequencing to determine the impact of FG-4592 on the composition and alterations of gut microbiota. The cloud plots show the alpha-diversity among the three groups by Ace and Shannon indices and observed OTUs (Figures 2A–C). Although we observed increased alpha-diversity in the DKD group over the Con and DKD-FG groups, there was no significant difference among the three (Figures 2A–C). However, principal coordinate analysis (PCoA) showing visualized beta-diversity suggest a significantly distinct microbial composition among these groups (Adonis for PCoA, R^2^ = 0.274, *p* < 0.001, Figures 2D,E and Supplementary Tables S4). As exhibited in Venn diagram overlaps, 422 of the 674 OTUs were shared by three groups; 14 OTUs, 122 OTUs, and 12 OTUs were specific for the Con, DKD, and DKD-FG groups, respectively (Figure 2F; Supplementary Table S5).

**FIGURE 3:**
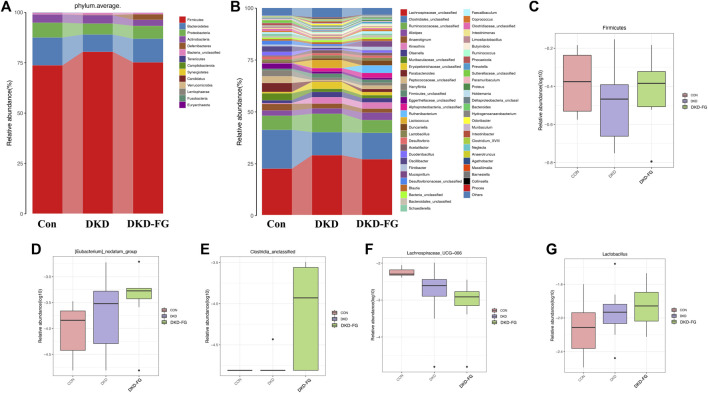
Composition of microbial communities at phylum **(A)** and genus **(B)** levels in Con, DKD, and DKD-FG groups. The Kruskal–Wallis rank-sum test was implemented to compare and identify significantly different bacteria at the phylum or genus level **(C–G)**. *p < 0.05, **p < 0.01, ***p < 0.001.

Analysis of relative abundance also suggests that the microbial structure of DKD-FG had altered more significantly than the Con and DKD groups. The average gut microbiota in all three groups were dominated by the phyla Firmicutes, Bacteroidetes*,* Proteobacteria, and Actinobacteria (accounting for more than 95% in all three groups, Figure 3A and Supplementary Table S6). Kruskal–Wallis rank-sum testing was used to compare and identify significantly different bacteria at the phylum and genus levels. The results revealed that the phylum Firmicutes had accumulated more in the DKD-FG group than in DKD (Figure 3C; Supplementary Table S7). Fusobacteria was the sole phylum existing in the gut microbiota in the DKD-FG group, but it did not exist in the gut microbiota of Con and DKD. Compared with the Con and DKD groups, there were 16 genera that were relatively accumulated and nine that were relatively depleted in DKD-FG. Among these, the relative abundance of genera *Clostridiales_unclassified [Eubacterium] nodatum* group and *Lactobacillus* were most significantly increased (Figures 3D,E,G, *p*-value < 0.001) in the DKD-FG group, while the relative abundance of genus *Lachnospiraceae UCG-006* was most significantly decreased (*p*-value < 0.001) in that group (Figure 3F; Supplementary Table S8, S9). All the aforementioned findings collectively demonstrate that FG-4592 can dramatically change the profiles and structures of gut microbiota in DKD mice.

### 3.4 FG-4592 alters the profiles of key OTUs and important functional pathways

**FIGURE 4:**
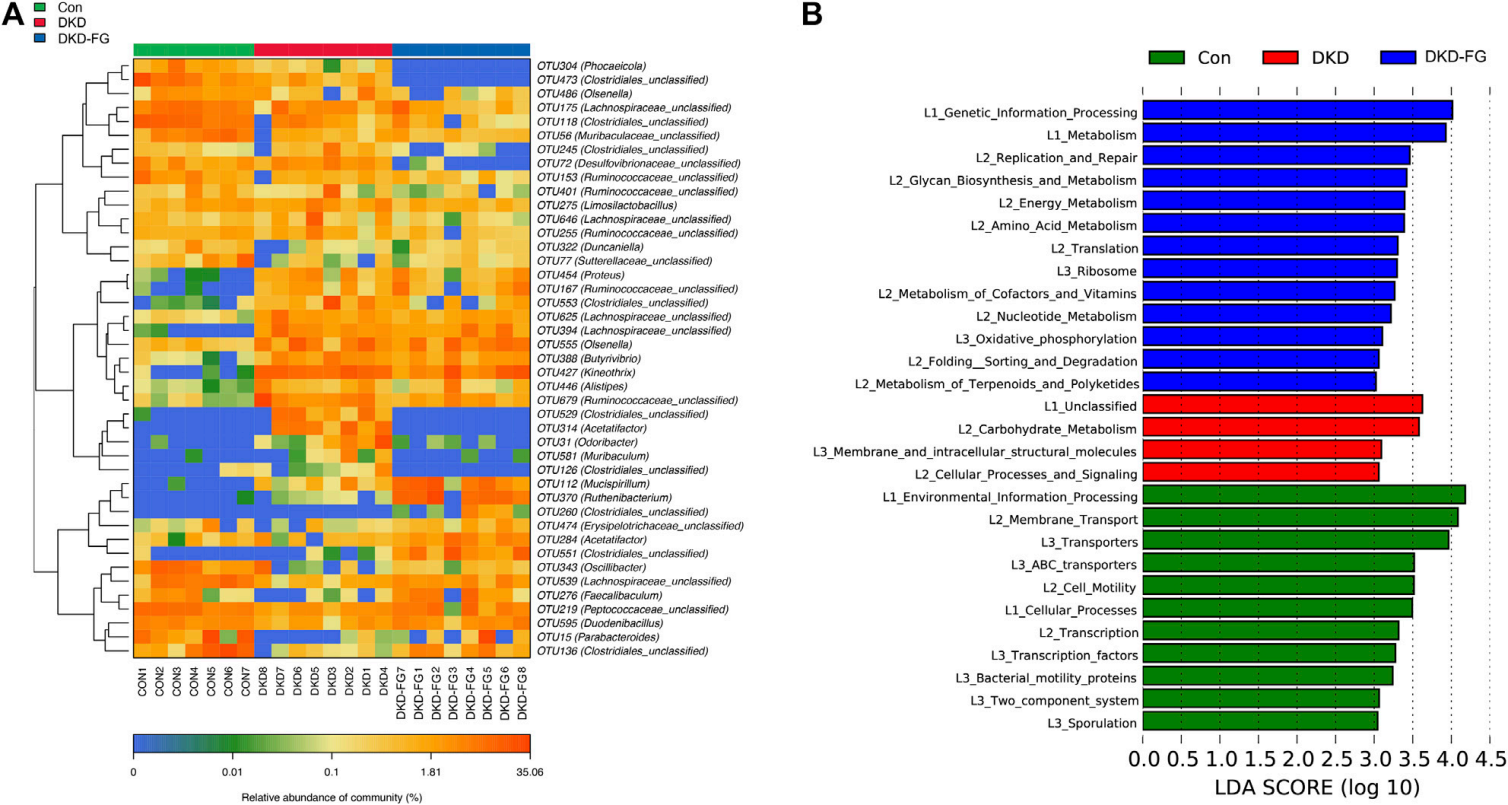
Distribution of key OTUs and enrichment of KEGG functional pathways among Con, DKD, and DKD-FG groups. Through the Wilcoxon rank-sum test, 43 OTUs with *p*-value > 0.05 and abundance >0.003% were considered key lineages for DKD and displayed as a heatmap **(A)**. Blue represents lower abundance; orange represents higher abundance. KEGG pathways indicated by LEfSe analysis with LDA score ≥3.0 and *p* ≤ 0.05 **(B)**. OTU, operational taxonomic units; KEGG, Kyoto Encyclopedia of Genes and Genomes; LEfSe, linear discriminate analysis and effect size; LDA, linear discriminant analysis. *p < 0.05, **p < 0.01, ***p < 0.001.

After we revealed that FG-4592 treatment induced dramatic alterations in the gut microbiota of DKD mice, we then focused on OTUs at the genus level and specific functional pathways. Kruskal–Wallis rank-sum testing showed that 249 OTUs at genus level were significantly different among the three groups. Among them, 67 OTUs were relatively accumulated in the DKD-FG group compared with those of the Con and DKD groups, while 48 OTUs were relatively depleted in the DKD-FG group (Figure 4A; Supplementary Table S10). Gut microbes with mean abundance larger than 0.003% and *p*-value lower than 0.05 through Wilcoxon testing were considered key OTUs. Some 43 OTUs were selected and presented as a heatmap, showing an apparently separated distribution among the Con, DKD, and DKD-FG groups (Figure 4A; Supplementary Table S11). According to the results of Kruskal–Wallis testing, 10 of 43 key OTUs were relatively depleted in the DKD-FG group compared to the Con and DKD groups, while 14 of 43 key OTUs were relatively depleted in the DKD-FG group (Supplementary Table S12). Among them, OTU118, 473, and 529 *(Clostridiales_unclassified)*, OTU72 *(Desulfovibrionaceae_unclassified)* and OTU304 *(Phocaeicola)* were the most significantly depleted (*p*-value <0.001) in the DKD-FG group than those in the Con and DKD, while OTU112 *(Mucispirillum)*, OTU551 and 260 *(Clostridiales_unclassified)*, OTU394 *(Lachnospiraceae_unclassified)*, and OTU388 *(Butyrivibrio)* were the most significantly accumulated (*p*-value <0.001) in the DKD-FG.

To explore the functional profiles among the Con, DKD, and DKD-FG groups, we performed a functional pathway analysis. Enrichment of the Kyoto Encyclopedia of Genes and Genomes (KEGG) metabolic pathways among the Con, DKD, and DKD-FG groups were predicted using PICRUSt. The KEGG metabolic pathways whose LDA >3.0 and *p*-value <0.05 were recognized as significantly different metabolic pathways. The functional pathways, including amino sugar and nucleotide sugar metabolisms, were significantly enhanced in the DKD-FG groups (Figure 4B; Supplementary Table S13). Taken together, FG-4592 can reconstruct the composition of key OTUs and significantly alter the functional state of gut microbiota.

### 3.5 FG-4592 induces unique profiles of plasma metabolomics in DKD mice

**FIGURE 5:**
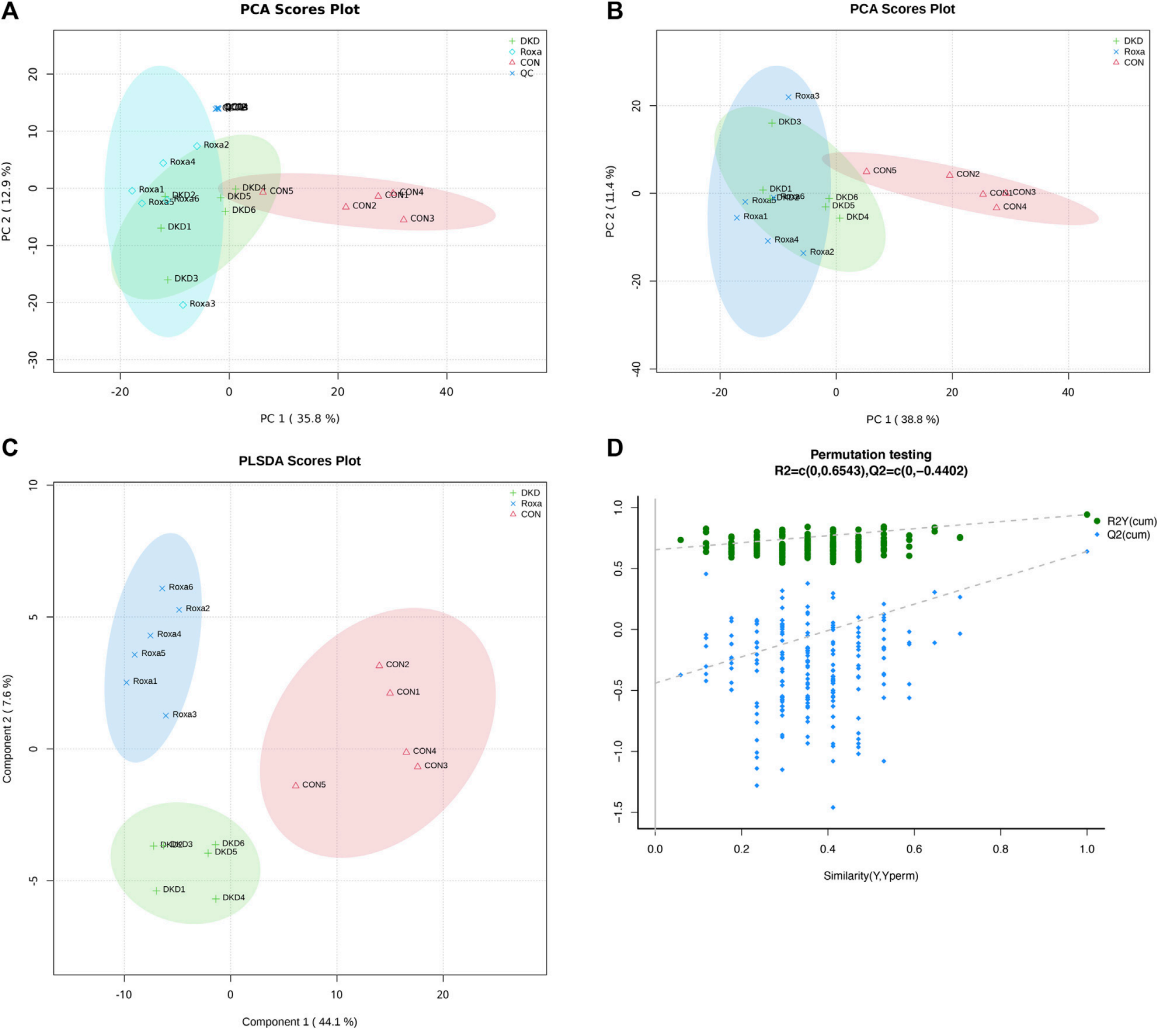
Validation of plasma metabolite disparity among Con, DKD, and DKD-FG groups. PCA score plots with **(A)** or without **(B)** internal QC; PLS-DA score plot **(C)**; **(D)** scatter plots of statistical validations obtained by 200s permutation tests. QC, quality control; PCA, principal component analysis; OPLS-DA, orthogonal partial least-squares discrimination analysis.

We have demonstrated that FG-4592 can relieve phenotypes in DKD mice and that this can be induced only in the presence of gut microbiota. Moreover, we have also revealed that the gut microbiota of DKD mice were dramatically reconstructed after FG-4592 consumption and selected the key OTUs. Therefore, we further employed plasma-untargeted metabolomic analysis to globally delineate the metabolic features of the Con, DKD, and DKD-FG groups to discover the underlying effects of FG-4592 and FG-4592-reconstructed gut microbiota on plasma metabolomics. Some 17 (five from Con, six from DKD, and six from DKD-FG) plasma samples were collected and subjected to ultra-performance liquid chromatography-mass spectrometry (UPLC-MS), identifying 272 and 368 metabolites in positive and negative ion modes, respectively (Supplementary Tables S14, S15). Multivariate statistical analyses were used to assess plasma metabolic characteristics. The stability of the sample collection and handling were evaluated by internal quality control (QC). PCA with internal QC provided an unsupervised and comprehensive view of the plasma samples and the excellent stability of the model. Significant separations were illustrated among the Con, DKD, and DKD-FG groups, with an acceptable explanatory value of the PCA model (cumulative R^2^X = 0.58, Figure 5A). PCA without internal QC showed similar results repeatedly (cumulative R^2^X = 0.50, Figure 5B). PLS-DA analysis was performed to maximally analyze the difference and confirmed marked altered plasma metabolite profiles among the three groups (R^2^Y = 0.94, Q^2^ = 0.64, Figure 5C). In 200s permutation testing, all R^2^ and Q^2^ values of permutated models were worse than the original model, indicating the better prediction ability and reliability of this model (Figure 5D). Therefore, we revealed that FG-4592 can reprogram the plasma metabolomics of DKD mice toward a unique orientation distinct from the Con and DKD groups.

### 3.6 FG-4592 reprograms the profiles of important differential metabolites

After discovering that FG-4592 can dramatically change the plasma metabolic features of DKD mice, we further tried to select the candidate metabolites that could participate in the mechanisms of FG-4592-induced DKD-relieving effects. By combining OPLS-DA analysis with one-way ANOVA or the Kruskal–Wallis test, we screened the 159 metabolites whose VIP score was >1 and *p*-value <0.05, and we defined them as important differential metabolites, including 45 metabolites in ESI + mode and 114 in ESI-mode (Supplementary Table S16). Among them, the expression level of 22 important differential metabolites was significantly increased in the DKD-FG group than in Con and DKD (Table 3). The expression level of propylene glycol stearate, isoswertisin 4′-glucoside, LysoPC(14:0/0:0), and LysoPC(16:1(9Z)/0:0) was significantly decreased in the DKD-FG group.

**TABLE 3:** Key OTUs relatively depleted or accumulated in DKD-FG group compared with Con and DKD groups.

	Depleted in the DKD-FG group						Accumulated in the DKD-FG group				
OTU	Con.mean	DKD.mean	Roxa.mean	*p*-value	Sig_mark	OTU	Con.mean	DKD.mean	Roxa.mean	*p*-value	Sig_mark
OTU118 (*Clostridiales_unclassified*)	0.04,348,471	0.00632,625	0.00441,238	<0.001	***	OTU112 (Mucispirillum)	<0.001	0.002069	0.02,727,125	<0.001	***
OTU473 (*Clostridiales_unclassified*)	0.03,512,914	0.004704	0	<0.001	***	OTU551 (Clostridiales_unclassified)	<0.001	4.70E-05	0.0199,555	<0.001	***
OTU72 (*Desulfovibrionaceae_unclassified*)	0.011273	0.00735,138	<0.001	<0.001	***	OTU394 (Lachnospiraceae_unclassified)	<0.001	0.009884	0.01,021,063	<0.001	***
OTU304 (*Phocaeicola*)	0.01,173,486	<0.001	0	<0.001	***	OTU388 (Butyrivibrio)	<0.001	0.007386	0.0075745	<0.001	***
OTU529 (*Clostridiales_unclassified*)	<0.001	0.0109,905	0	<0.001	***	OTU260 (Clostridiales_unclassified)	0	0	0.00308,975	<0.001	***
OTU343 (*Oscillibacter*)	0.02,628,271	0.006165	0.00159,275	0.00129,254	**	OTU370 (Ruthenibacterium)	<0.001	<0.001	0.03,514,213	0.00123,138	**
OTU153 (*Ruminococcaceae_unclassified*)	0.01,494,386	0.00238,313	0.00231,888	0.00454,984	**	OTU276 (Faecalibaculum)	0.00810,286	<0.001	0.01,183,925	0.00410,363	**
OTU255 (*Ruminococcaceae_unclassified*)	0.00558,829	0.00317,463	0.00135,538	0.00185,767	**	OTU284 (Acetatifactor)	0.00274	0.00330,313	0.01,142,988	0.00544,254	**
OTU401 (*Ruminococcaceae_unclassified*)	0.00467,171	0.0033835	<0.001	0.00291,318	**	OTU167 (Ruminococcaceae_unclassified)	<0.001	0.00326,063	0.00952,025	0.00300,736	**
OTU245 (*Clostridiales_unclassified*)	0.00148,429	0.00282,338	<0.001	0.00624,378	**	OTU454 (*Proteus*)	<0.001	0.004442	0.00577,413	0.00233,777	**
OTU126 (*Clostridiales_unclassified*)	<0.001	<0.001	0	0.00558,901	**						
OTU486 (*Olsenella*)	0.01,077,143	0.00533,775	0.00107,763	0.02,640,195	*						
OTU275 (*Limosilactobacillus*)	0.00745,371	0.0053945	0.00299,775	0.02,768,245	*						
OTU646 (*Lachnospiraceae_unclassified*)	0.00285,586	0.0045055	<0.001	0.01,502,879	*						

OTU, operational taxonomic units; DKD, diabetic kidney disease; Con, control; Sig, significance; *p < 0.05, **p < 0.01, ***p < 0.001.

**FIGURE 6:**
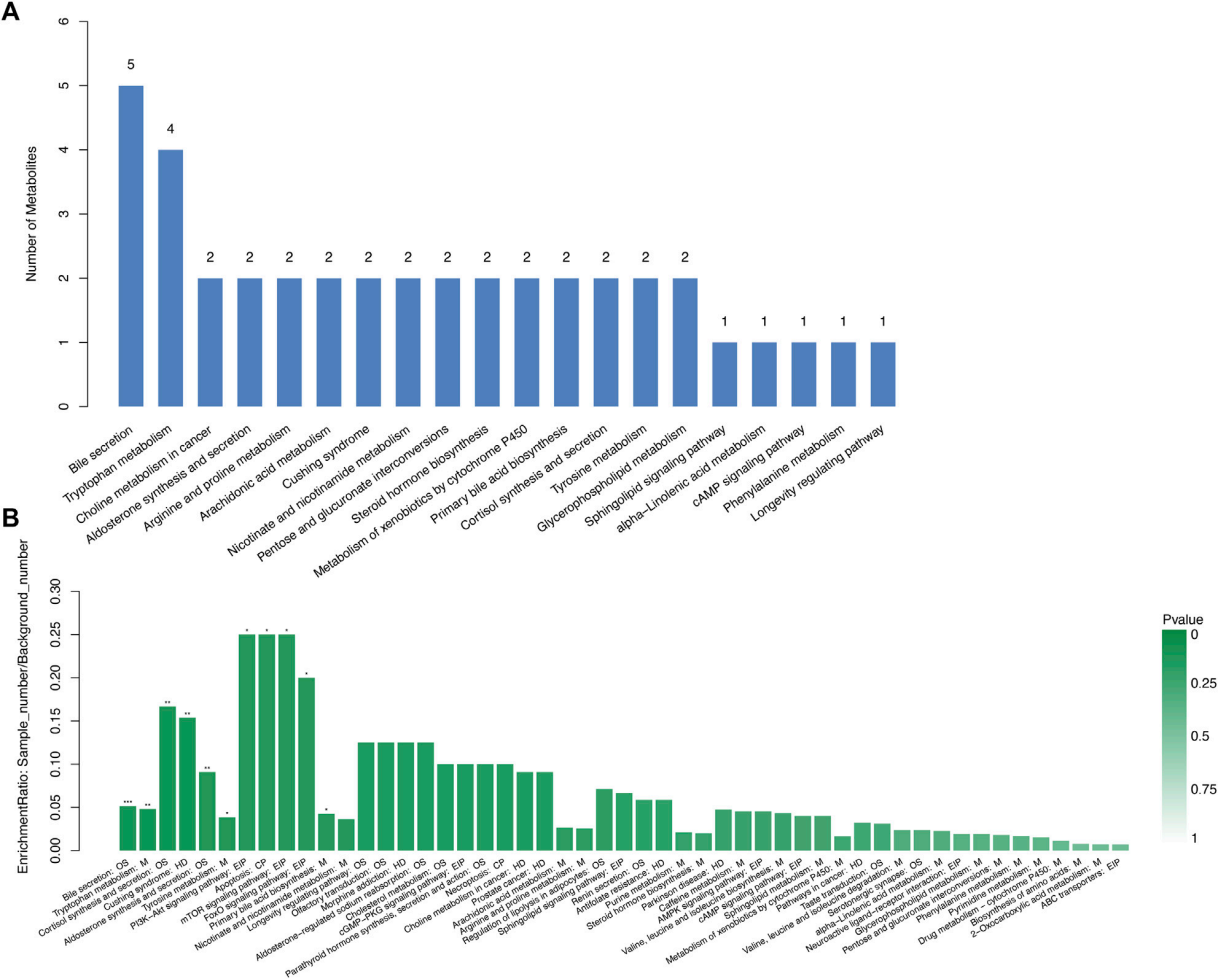
Advanced functional metabolic pathways analysis of Con, DKD, and DKD-FG groups. Top 20 metabolic pathways ranked by the number of important differential metabolites they contained **(A)**. KEGG enrichment analysis of significantly enriched metabolic pathways **(B)**. OS, organismal system; M, metabolism; HD, human disease; EIP, environmental information processing; CP, cellular processing.

By searching the KEGG/HMDB database, we obtained the pathways in which 159 important differential metabolites are involved. Among the top 20 enriched metabolic pathways ranked by the number of important differential metabolites they contained, bile secretion contained the largest number of metabolites (five), followed by the tryptophan metabolism containing four types of metabolites (Figure 6A; Supplementary Table S17). KEGG pathway enrichment analysis annotated important differential metabolites to their involved metabolic pathways and evaluated their degree and significance of enrichment (Figure 6B; Supplementary Table S18). Xanthurenic acid, the metabolite most significantly accumulated in the DKD-FG group, belongs to and has a great impact on tryptophan metabolism (Figures 6A,B). Furthermore, 17-beta-estradiol-3-glucuronide and 6-dehydrotestosterone glucuronide belong to both bile secretion and pentose and glucuronate interconversions (Figures 6A,B). Traumatic acid belongs to alpha-linolenic acid metabolism, which is related to lipid metabolism.

### 3.7 FG-4592 reprograms plasma metabolomics in DKD mice via gut microbiota reconstruction

**FIGURE 7:**
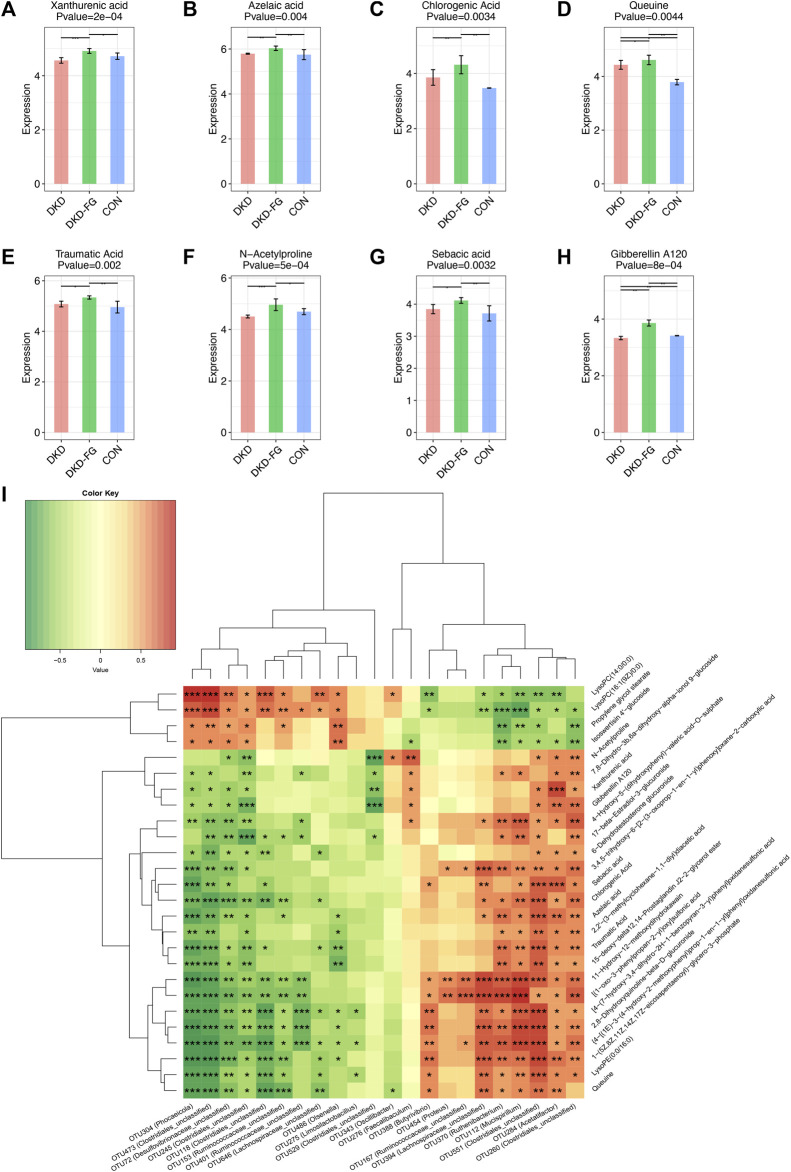
Metabolic alterations of mice models based on untargeted metabolomic detection and Spearman’s correlation analysis between important differential metabolites and key OTUs. Boxplots showing the comparison of the relative expression level of eight important differential metabolites among three groups **(A–H)**. Spearman’s correlation relationship between important differential metabolites and key OTUs presented as a heatmap **(I)**. *p < 0.05; **p < 0.01; ***p < 0.001; ****p < 0.0001.

To illustrate the underlying connection between gut microbiota and plasma metabolism and their role in FG-4592-induced alleviation of DKD, we implemented gut microbiota-untargeted plasma metabolomics combining Spearman’s correlation analysis (Figure 7; Supplementary Table S19). As shown in Figure 7, important differential metabolites revealed a dramatic correlation with gut microbiota. Xanthurenic acid (XA) significantly accumulated in the DKD-FG group more than in the DKD group (Figure 7A). It was negatively correlated with the quantity of OTU304 (*Phocaeicola*), 72 (*Desulfovibrionaceae-unclassified*), 473, 529, and 245 (*Clostridiales-unclassified*) and positively correlated with the quantity of OTU 284 (*Acetatifactor*), 551, and 260 (*Clostridiales-unclassified*) (Figure 7I). Azelaic acid was relatively increased in the DKD-FG group (Figure 7B; Table 4) and positively associated with OTU284 (*Acetatifactor*), 551, and 260 (*Clostridiales_unclassified*), OTU112 (*Mucispirillum*), OTU370 (*Ruthenibacterium*), and OTU394 (*Lachnospiraceae_unclassified*) and was negatively associated with OTU486 (*Olsenella*), OTU153 (*Ruminococcaceae_unclassified*), OTU304 (*Phocaeicola*), 72 (*Desulfovibrionaceae_unclassified*), and 473 and 245 (*Clostridiales_unclassified*), which were all depleted in the DKD-FG group (Figure 7I). Chlorogenic acid was also present in a correlation pattern similar to microbiota and metabolomics (Figure 7C; Table 4). Queuine significantly accumulated in the DKD-FG-4592 group (Figure 7D; Table 2) and was positively associated with OTU 284 (*Acetatifactor*), 551 (*Clostridiales_unclassified*), OTU112 (*Mucispirillum*), OTU370 (*Ruthenibacterium*), and OTU394 (*Lachnospiraceae_unclassified*) and was negatively correlated with OTU646 (*Lachnospiraceae_unclassified*), OTU153 (*Ruminococcaceae_unclassified*), OTU304 (*Phocaeicola*), 72 (*Desulfovibrionaceae_unclassified*), 118, 473, and 245 (*Clostridiales_unclassified*), which were all depleted in the DKD-FG-4592 group (Figure 7I).

**TABLE 4:** The important differential metabolites that significantly increased in the serum of DKD-FG group when compared with those in Con and DKD groups.

Metabolite	Mean_CON	Mean_DKD	Mean_DKD-FG	group_Pvalue	VIP score	Sig. mark
Xanthurenic acid	4.7227094	4.565622	4.91566317	0.00015275	1.70802659	***
N-Acetylproline	4.6928868	4.503325	4.9606255	0.00053901	1.90568564	***
Gibberellin A120	3.414465	3.3308265	3.8608245	0.00081067	2.3079743	***
1-(5Z,8Z,11Z,14Z,17Z-eicosapentaenoyl)-glycero-3-phosphate	5.9383608	6.171173	6.40616617	<0.001	1.08415124	***
[4-(7-hydroxy-3,4-dihydro-2H-1-benzopyran-3-yl)phenyl]oxidanesulfonic acid	5.4468924	5.86727133	6.31059033	<0.001	1.46385773	***
2,2'-(3-methylcyclohexane-1,1-diyl)diacetic acid	4.786976	4.77600117	5.21510867	0.00478934	1.62462567	**
Traumatic Acid	4.9573082	5.07914267	5.3384885	0.00204248	1.13203723	**
Sebacic acid	3.7131192	3.84675867	4.11544617	0.00322108	1.13645336	**
15-deoxy-delta12,14-Prostaglandin J2-2-glycerol ester	3.7703884	3.93459517	4.69630067	0.00172144	2.08032332	**
3,4,5-trihydroxy-6-[2-(3-oxoprop-1-en-1-yl)phenoxy]oxane-2-carboxylic acid	3.9885252	4.23616067	4.72613083	0.00453066	1.57606692	**
LysoPE(0:0/16:0)	3.016404	3.665625	4.7906755	0.00523895	2.26221512	**
Azelaic acid	5.749735	5.7912655	6.0329955	0.00397092	1.23717466	**
Chlorogenic Acid	3.471726	3.8550995	4.315934	0.00339442	1.87213691	**
Queuine	3.788067	4.431797	4.6134105	0.00440867	1.1112149	**
2,8-Dihydroxyquinoline-beta-D-glucuronide	4.905663	5.943125	6.2545115	0.00101905	1.5291593	**
[(1-oxo-3-phenylpropan-2-yl)oxy]sulfonic acid	2.396237	4.060791	4.6215015	0.00187144	1.85133743	**
11-Hydroxy-12-methoxydihydrokawain	2.344633	4.102998	4.3788945	0.00154427	1.5069721	**
{4-[(1E)-3-(4-hydroxy-2-methoxyphenyl)prop-1-en-1-yl]phenyl}oxidanesulfonic acid	2.572547	3.8432295	4.7623785	0.00121255	2.12240165	**
7,8-Dihydro-3b,6a-dihydroxy-alpha-ionol 9-glucoside	3.508943	3.474872	4.0035435	0.01086485	1.69256086	*
17-beta-Estradiol-3-glucuronide	2.930248	3.602213	4.1427195	0.03257588	1.36497413	*
6-Dehydrotestosterone glucuronide	3.627624	4.0425965	4.407909	0.03257588	1.39016704	*
4-Hydroxy-5-(dihydroxyphenyl)-valeric acid-O-sulphate	3.704804	3.732092	4.3468195	0.02144028	1.52296828	*

DKD, diabetic kidney disease; Con, control; VIP, variable importance in projection; Sig, significance; *p < 0.05, **p < 0.01, ***p < 0.001.

## 4 Discussion

In this study, we evaluated the effects of FG-4592 on DKD clinical signs in both humans and mice and analyzed the alterations and correlation of both gut microbiota and plasma metabolism after FG-4592 treatment in DKD mice, thus guiding FG-4592 administration in DKD patients with renal anemia. The major finding of our study was that FG-4592 application of at least 3–6 months can effectively relieve the clinical phenotypes of DKD in humans by decreasing SBP and serum Cr levels and improving eGFR, indicating the potential preventative effects of DKD progression. We also observed similar global improving effects in DKD mice, including elevated body weight and decreased urine protein after treatment with FG-4592, indicating the potential protection effects of FG-4592 in DKD. We also revealed that the DKD-relieving effects were only induced by FG-4592 in the male group.

As a HIF stabilizer, it has been reported that FG-4592 can dramatically alter the functional and metabolic state of the gastrointestinal system and gut microbiota. We focused on the influence of FG-4592 on gut microbiota and systemic plasma metabolomics, and subsequently the effects on DKD phenotypes in a mice model. We revealed the dramatically changed gut microbiota and plasma metabolomics of DKD mice and listed the important significantly different OTUs and plasma metabolites in Tables 2 and 3. Among these, xanthurenic acid (XA), the 8-hydroxylated analog of kynurenic acid, is reported as higher in schizophrenia patients with obesity (BMI≥25) than patients whose BMI ≤25, which may act as the metabolite associated with increased body weight after FG-4592 treatment (Oxenkrug et al., 2019). In our study, XA was significantly accumulated in the DKD-FG-4592 group over the DKD group. FG-4592 reconstructs the composition of gut microbiota by decreasing the abundance of OTU304 (*Phocaeicola*), 72 (*Desulfovibrionaceae_unclassified*), 473, 529, and 245 (*Clostridiales_unclassified*), which were negatively correlated with the level of XA, and increasing the abundance of OTU284 (*Acetatifactor*), and 551 and 260 (*Clostridiales-unclassified*), which were positively correlated with the level of XA (Table 2; Figure 5). The unique composition collectively increased the level of XA in the FG-4592 group than the DKD and Con groups, which further increase the body weight of FG-4592-treated DKD mice. However, XA is a key metabolite of the kynurenine pathway, known to be associated with the development of type 2 diabetes (Law and Zhang, 2017; Song et al., 2017).

Furthermore, azelaic acid and chlorogenic acid are also reported to harbor antioxidant effects which are widely topically used in acne and other inflammatory skin diseases (Markiewicz-Tomczyk et al., 2022; Szabo et al., 2022). In our study, azelaic acid was relatively increased in the DKD-FG group. According to the results of microbiota-metabolomics combing correlation analysis, azelaic acid was positively associated with OTU284 (*Acetatifactor*), 551 and 260 (*Clostridiales_unclassified*), OTU112 (*Mucispirillum*), OTU370 (*Ruthenibacterium*), and OTU394 (*Lachnospiraceae_unclassified*), which were all accumulated in the DKD-FG group, and was negatively associated with OTU486 (*Olsenella*), OTU153 (*Ruminococcaceae_unclassified*), OTU304 (*Phocaeicola*), 72 (*Desulfovibrionaceae_unclassified*), 473, and 245 (*Clostridiales_unclassified*), which were all depleted in the DKD-FG group. Chlorogenic acid also presented a similar correlation pattern between microbiota and metabolomics. In summary, we reasonably speculate that FG-4592 alters the structure of gut microbiota to increase the level of azelaic acid, which plays an important role in anti-inflammation. The concrete pathogenetic mechanism of proteinuria is complicated; it is generally involved in interstitial inflammation and renal fibrosis (Abbate et al., 2006). FG-4592 may potentially exert a proteinuria-alleviating effect by reconstructing gut microbiota to regulate specific anti-inflammatory metabolites, thus preventing DKD progression.

Interestingly, we found that phylum Deferribacteres and genus *Ruthenibacterium* were especially enriched in the DKD-FG group. As divalent iron is better absorbed by the human body than trivalent iron, many species in Deferribacteres were considered to be associated with the reducing power of the ferric ion (Tamazawa et al., 2017), which might also be one of the effects of FG-4592 on improving anemia. *R. lactatiformans* is the most famous species in genus *Ruthenibacterium* and is increased in relapsing remitting multiple sclerosis (RRMS) patients (Cox et al., 2021). Meanwhile, Mediterranean diet subjects had a lower abundance of *Ruthenibacterium*, together with lower levels of branched-chain fatty acid. We inferred that the enrichment of *Ruthenibacterium* might be by influencing a fatty acid metabolism to improve the DKD phenotype (Cox et al., 2021).

Queuine is a pyrrolopyrimidine-containing analog of guanine (Figure 1) that is exclusively synthesized by bacteria and found in most eukaryotes, including humans who acquire queuine from their own gut microbiota and a diet that contains this bacterial-derived molecule (Fergus et al., 2015; Richard et al., 2021). DKD progression is associated with low-grade inflammation and mitochondrial dysfunction (Zhan et al., 2015; Ducasa et al., 2019; Na et al., 2021; Mitrofanova et al., 2022). Queuine is reported to be involved in the recovery of mitochondrial dysfunction and has a protection effect in neurodegeneration diseases (Zheng et al., 2022). In our study, queuine is significantly accumulated in the DKD-FG group, which has protection effects against mitochondrial disturbance in DKD mice and improves DKD phenotypes. Furthermore, considering that the sole origin of queuine is intestinal bacteria, the correlation between gut microbiota and systemic metabolomics showed that queuine is positively associated with OTU284 (*Acetatifactor*), 551 (*Clostridiales-unclassified*), OTU112 (*Mucispirillum*), OTU370 (*Ruthenibacterium*), and OTU394 (*Lachnospiraceae-unclassified*), which were all accumulated in the DKD-FG group. Queuine is negatively correlated with OTU646 (*Lachnospiraceae-unclassified*), OTU153 (*Ruminococcaceae-unclassified*), OTU304 (*Phocaeicola*), 72 (*Desulfovibrionaceae-unclassified*), 118, 473, and 245 (*Clostridiales-unclassified*), which were all depleted in the DKD-FG group. Therefore, FG-4592 remodels the gut microbiota to increase the plasma level of queuine, which can partially recover mitochondrial dysfunction to improve the DKD phenotypes.

Intriguingly, according to the clinical data, we found that there is significant sex disparity in the pharmacological effects of FG-4592: it can only relieve DKD in males, presented as significantly lower SBP, DBP, and serum Cr, and higher serum Hb and eGFR. To our knowledge, ours is the first research to discuss the sex disparity of FG-4592 effects. Although such disparity may be due to the relatively smaller size of our cohort, it still strongly encourages us to expand the size of the observational cohort to further determine the specific effects of FG-4592 in different sexes.

There are some limitations to this study. We did not collect fecal samples from DKD patients for microbial community analysis to demonstrate the findings in animal experiments. We were unable to accurately locate which species play a crucial role in FG-4592 treatment of DKD, and the related molecular mechanisms were unclear. Our findings need to be further validated in order to provide new therapeutic targets for DKD, such as using specific probiotics to increase the efficacy of FG-4592.

Taken together, our study first revealed the DKD-alleviating effects of FG-4592 in both human and mouse models and delineated the profiles of gut microbiota and plasma metabolomics induced by FG-4592 treatment in an animal experiment. We discovered that FG-4592 can reconstruct gut microbiota to increase the level of beneficial metabolites, subsequently improving the DKD phenotypes. On the one hand, our findings may provide a theoretical foundation for expanding the range of FG-4592 applications which may be not limited to the treatment of renal anemia. On the other hand, we extracted the valuable differential metabolites regulated by FG-4592 which play an important role in DKD improvement. More advanced studies on the effects of these metabolites in DKD may be necessary to decipher the underlying pathogenetic mechanism. Moreover, specifically regulating such metabolites may also provide novel targets for DKD management in clinical practice.

## Data Availability

The datasets presented in this study can be found in online repositories. The names of the repository/repositories and accession number(s) can be found below: https://www.ncbi.nlm.nih.gov/ (accession numbers PRJNA948870; PRJNA876080, PRJNA881044). https://www.ebi.ac.uk/metabolights/ (accession numbers MTBLS7554; MTBLS6040).
