# Supplementary figures and images for: FG-4592 relieves diabetic kidney disease severity by influencing metabolic profiles via gut microbiota reconstruction in both human and mouse models
Source: Front Physiol. 2023 Aug 15;14:1195441. doi: 10.3389/fphys.2023.1195441 (PMC10465800; doi:10.3389/fphys.2023.1195441)

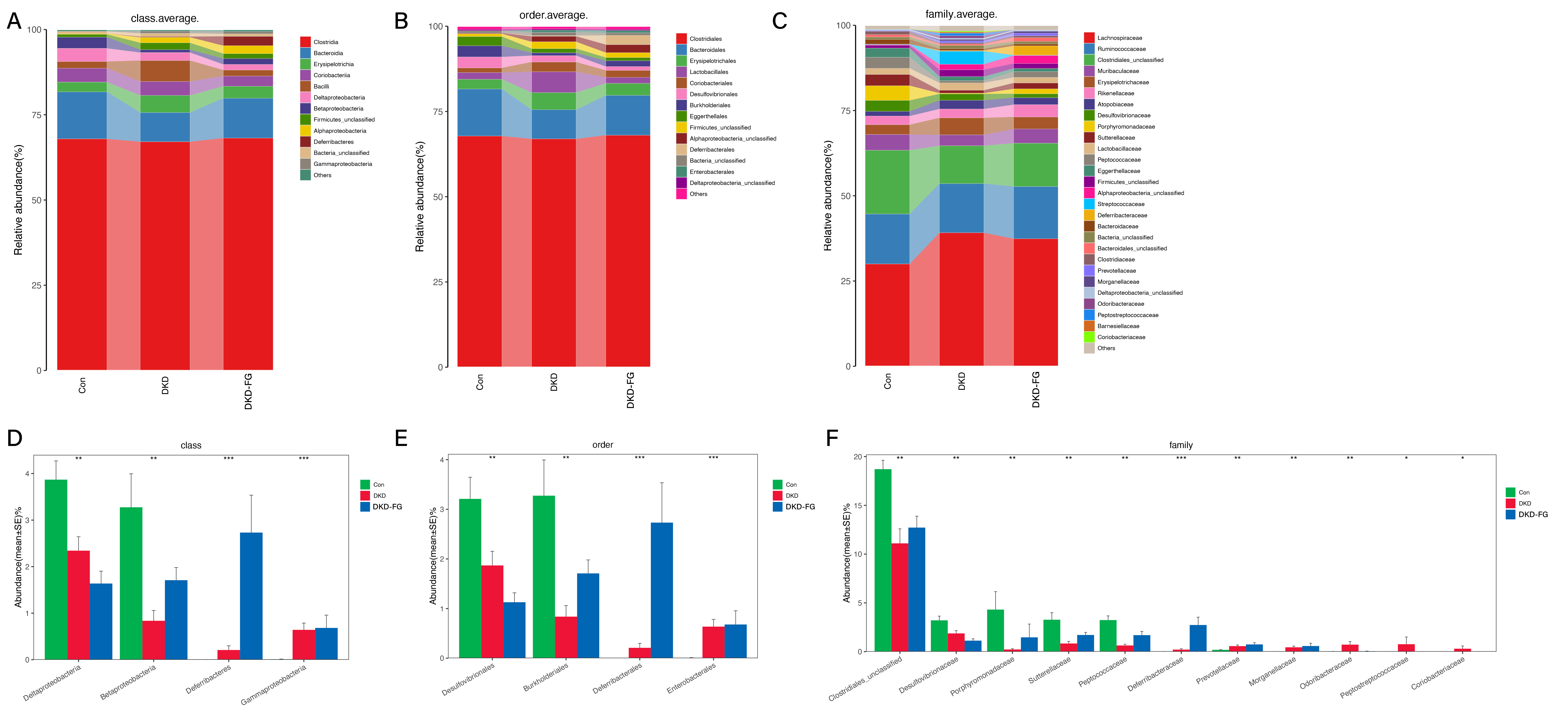

Supplement: Supplementary file 1 [file Image3.TIF]

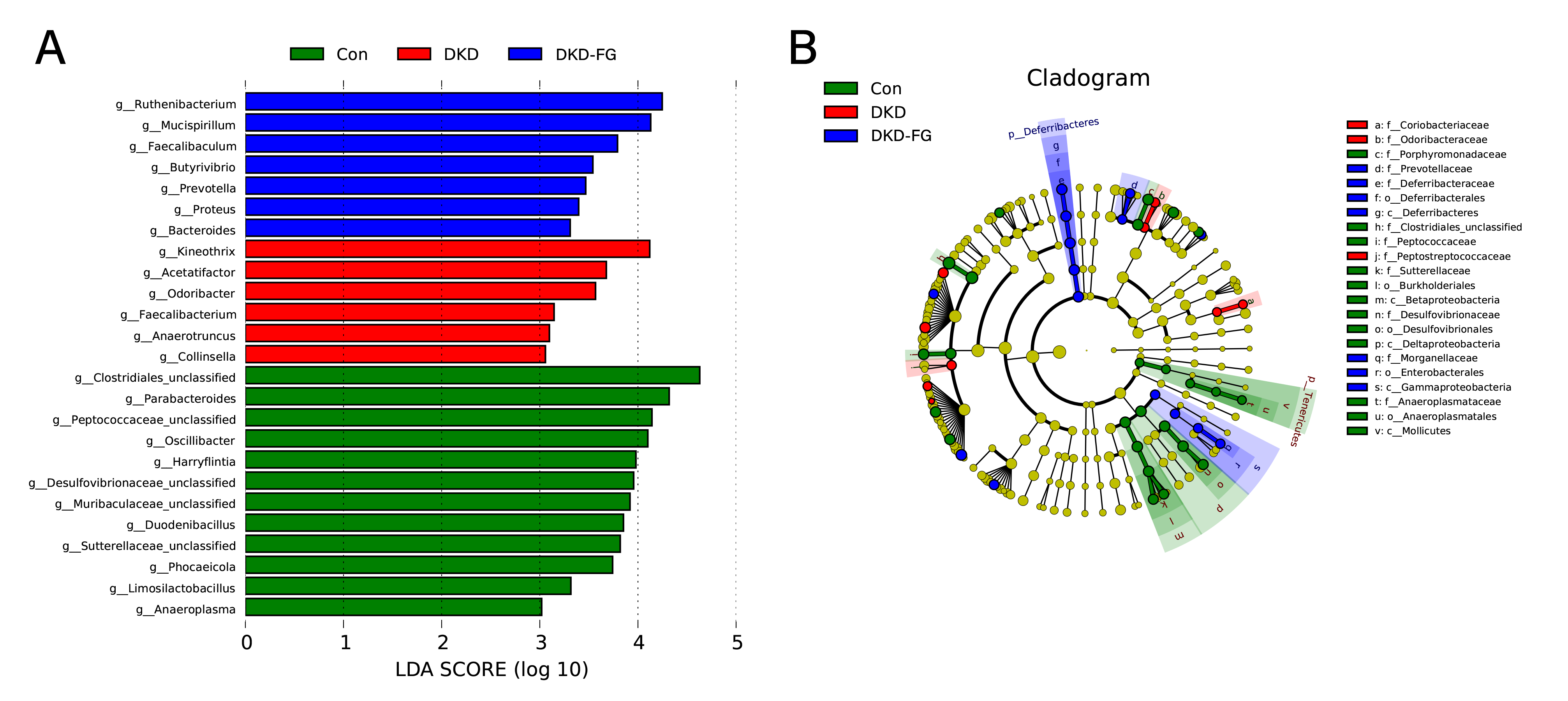

Supplement: Supplementary file 2 [file Image4.TIF]

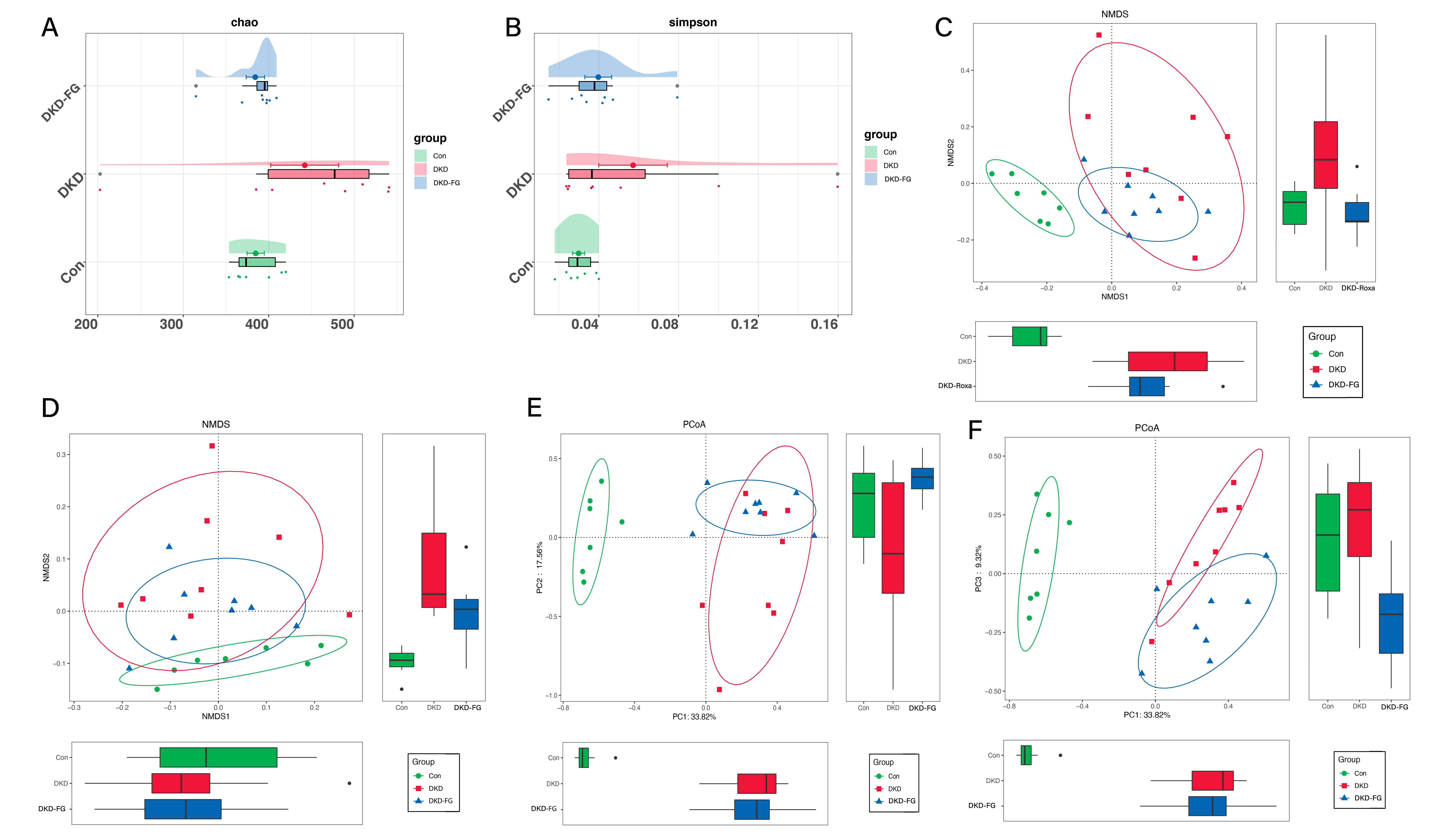

Supplement: Supplementary file 4 [file Image2.TIF]

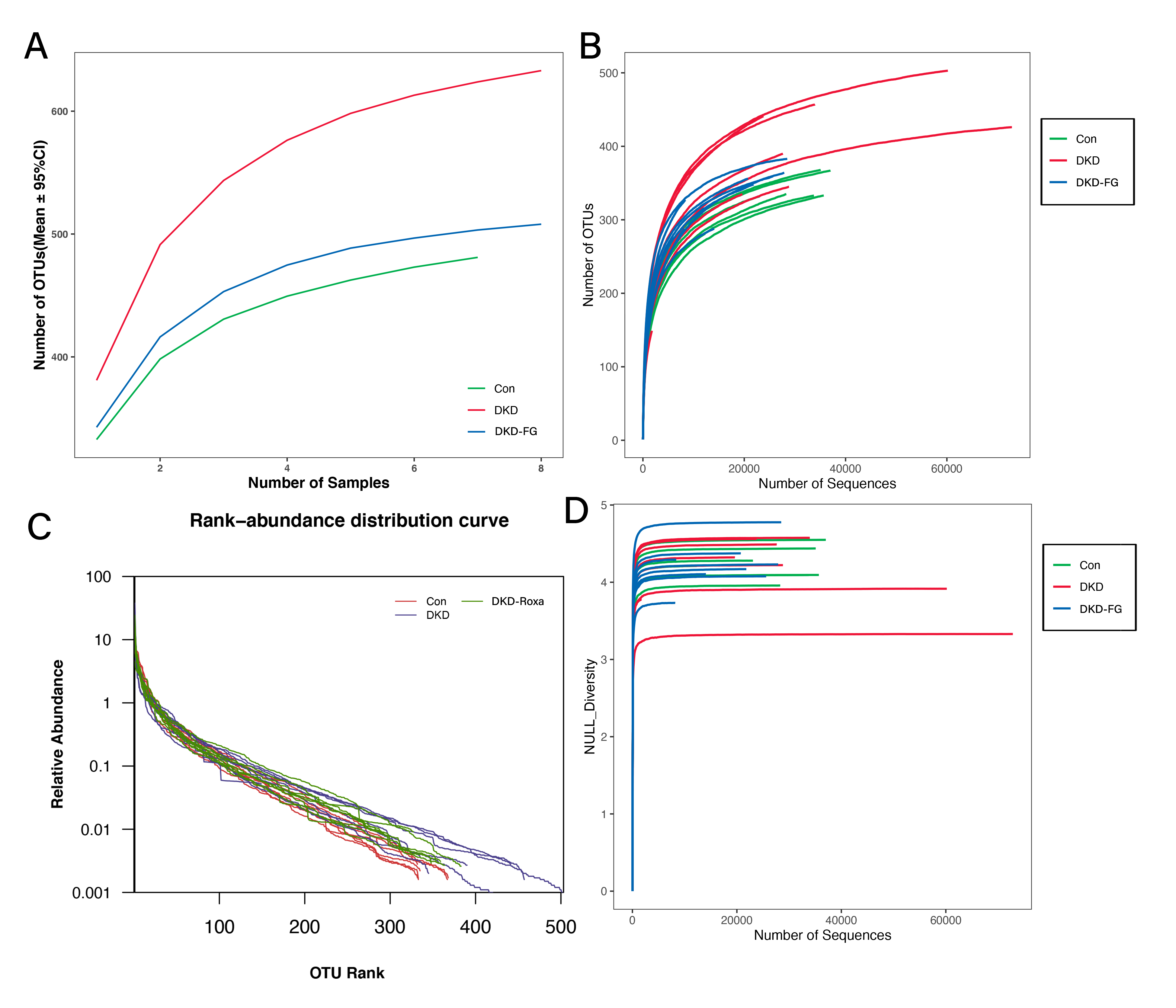

Supplement: Supplementary file 5 [file Image1.TIF]

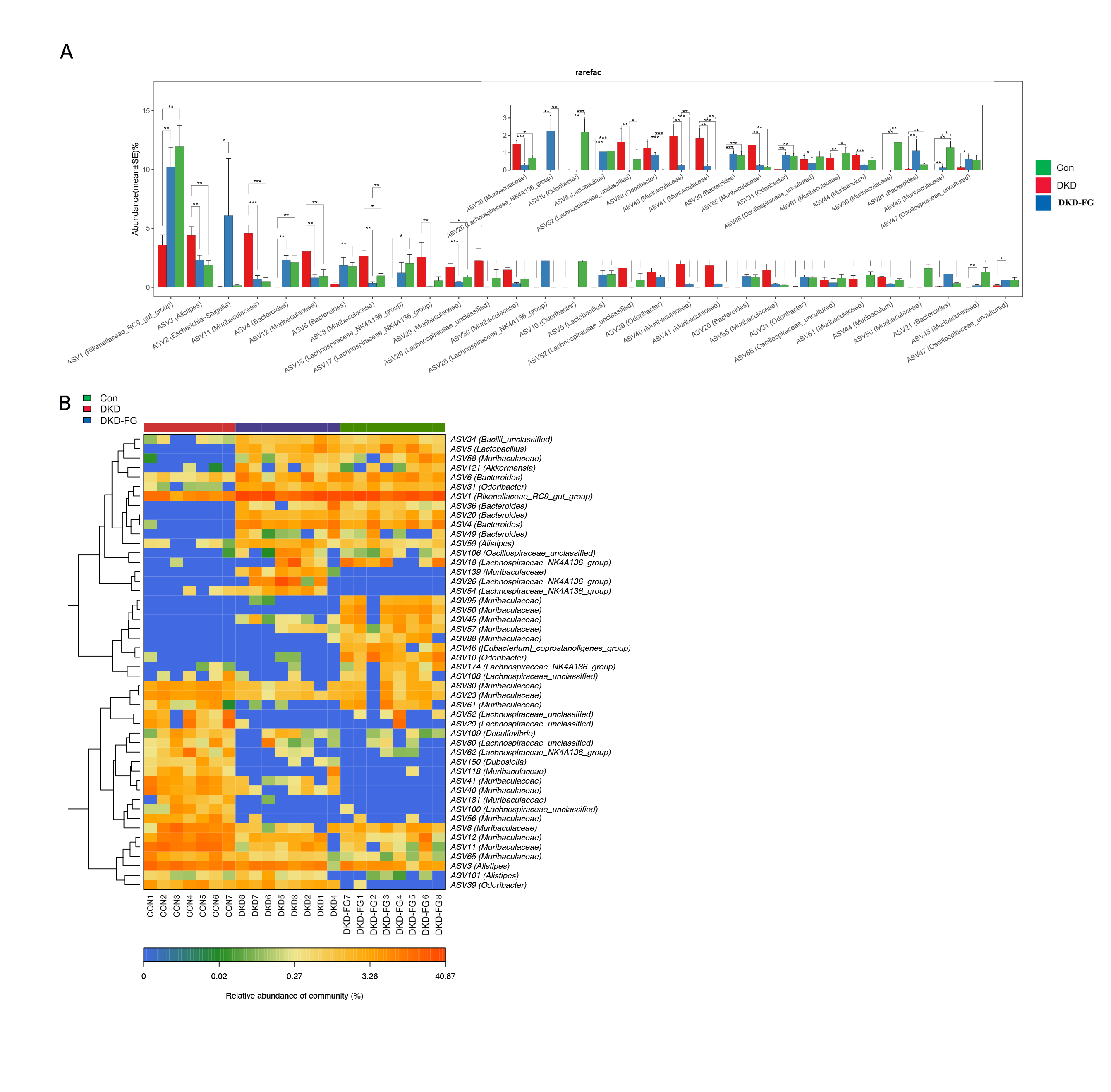

Supplement: Supplementary file 7 [file Image5.TIF]
