# Supplementary material for: FG-4592 relieves diabetic kidney disease severity by influencing metabolic profiles via gut microbiota reconstruction in both human and mouse models
Source: Front Physiol. 2023 Aug 15;14:1195441. doi: 10.3389/fphys.2023.1195441 (PMC10465800; doi:10.3389/fphys.2023.1195441)
Supplement: Supplementary file 3 [file DataSheet1.ZIP › Supplemental tables/Table S21.docx]

**TABLE S21.1 Demographic characteristics of male DKD patients combined renal anemia treated with FG-4592 group and control group at the beginning.**

|  | FG-4592 group (n=24) | Control group  (n=18) | P value |
| --- | --- | --- | --- |
| Age | 54.29±7.58 | 56.22±12.81 | 0.545 |
| Height(cm) | 171.5(167.5-175.0) | 167(167-171.25) | 0.046 |
| Body weight (Kg) | 72.0(62.3-81.5) | 72.0(72.0-76.5) | 0.431 |
| SBP (mmHg) | 135(123-152) | 140(134-151) | 0.408 |
| DBP (mmHg) | 81(78-87) | 78(72-91) | 0.315 |
| Hb(g/L) | 91.54±12.01 | 92.17±8.69 | 0.853 |
| Ghb (%) | 6.2(5.8-6.7) | 6.2(5.8-7.0) | 0.98 |
| Cr (μmol/L) | 239(156-392) | 257(198-355) | 0.485 |
| eGFR  (mL/min/l.73m^2^) | 24.650(13.925-44.250) | 21.345(15.990-29.668) | 0.509 |
| Alb (g/L) | 32.36±6.84 | 30.06±6.21 | 0.27 |
| 24hpro (g) | 5.00(1.55-8.93) | 5.22(3.29-7.87) | 0.722 |

**TABLE S21.2 Demographic characteristics of male DKD patients combined renal anemia treated with FG-4592 group and control group after 3-6 months follow-up period.**

|  | FG-4592 group (n=24) | Control group  (n=18) | P value |
| --- | --- | --- | --- |
| Age | 54.58±7.43 | 56.33±12.83 | 0.581 |
| Height(cm) | 171.5(167.5-175.0) | 167(167-171.25) | 0.046 |
| Body weight (Kg) | 72.0(62.3-81.5) | 72.0(72.0-76.5) | 0.431 |
| SBP (mmHg) | 138(129-145) | 152(136-165) | 0.003 |
| DBP (mmHg) | 81.50±9.24 | 87.94±11.29 | 0.049 |
| Hb(g/L) | 102.50(91.50-112.25) | 87.50(82.25-96.00) | 0.006 |
| Ghb (%) | 6.15(5.65-7.08) | 6.59(5.75-7.10) | 0.638 |
| Cr (μmol/L) | 231.50(161.25-371.00) | 333.35(233.25-501.25) | 0.019 |
| eGFR  (mL/min/l.73m^2^) | 25.500(15.225-39.750) | 14.562(9.880-24.912) | 0.011 |
| Alb (g/L) | 31.68±6.17 | 31.13±6.52 | 0.785 |
| 24hpro (g) | 4.07(3.01-7.71) | 5.30(4.15-7.65) | 0.286 |
