# Supplementary material for: FG-4592 relieves diabetic kidney disease severity by influencing metabolic profiles via gut microbiota reconstruction in both human and mouse models
Source: Front Physiol. 2023 Aug 15;14:1195441. doi: 10.3389/fphys.2023.1195441 (PMC10465800; doi:10.3389/fphys.2023.1195441)
Supplement: Supplementary file 3 [file DataSheet1.ZIP › Supplemental tables/Table S20.docx]

**TABLE S20.1 Demographic characteristics of female DKD patients combined renal anemia treated with FG-4592 group and control group at the beginning.**

|  | FG-4592 group (n=16) | Control group (n=15) | P value |
| --- | --- | --- | --- |
| Age | 51.33±11.04 | 52.53±11.21 | 0.782 |
| Height(cm) | 69.33±10.10 | 70.37±3.75 | 0.401 |
| Body weight (Kg) | 70(60-76) | 72(69-72) | 0.8 |
| SBP (mmHg) | 140(130-150) | 136(130-166) | 0.682 |
| DBP (mmHg) | 82.07±10.77 | 83.13±16.25 | 0.991 |
| Hb(g/L) | 90.40±9.24 | 93.53±6.70 | 0.329 |
| Ghb (%) | 6.10(5.80-7.50) | 6.67(5.86-8.30) | 0.412 |
| Cr (μmol/L) | 263.33±131.68 | 251.08±117.90 | 0.646 |
| eGFR  (mL/min/l.73m^2^) | 18.70(14.30-41.00) | 17.09(12.89-40.82) | 0.861 |
| Alb (g/L) | 34.867±7.22 | 31.773±5.63 | 0.14 |
| 24hpro (g) | 4.45±3.42 | 4.79±3.40 | 0.824 |

**TABLE S20.2 Demographic characteristics of female DKD patients combined renal anemia treated with FG-4592 group and control group after 3-6 months follow-up period.**

|  | FG-4592 group (n=16) | Control group  (n=15) | P value |
| --- | --- | --- | --- |
| Age | 51.75±10.54 | 52.73±11.29 | 0.804 |
| Height(cm) | 69.33±10.10 | 70.37±3.75 | 0.401 |
| Body weight (Kg) | 70(60-76) | 72(69-72) | 0.8 |
| SBP (mmHg) | 139(123-145) | 137(125-154) | 0.83 |
| DBP (mmHg) | 79.63±8.79 | 80.80±9.87 | 0.728 |
| Hb(g/L) | 92.50±18.70 | 96.67±13.86 | 0.489 |
| Ghb (%) | 6.2(5.6-6.5) | 6.3(5.5-7.8) | 0.299 |
| Cr (μmol/L) | 253.50(165.75-465.75) | 336.00(217.00-540.00) | 0.52 |
| eGFR  (mL/min/l.73m^2^) | 17,45(8.80-27.93) | 13.81(7.00-20.75) | 0.379 |
| Alb (g/L) | 32.53±6.95 | 31.62±6.53 | 0.71 |
| 24hpro (g) | 2.06(0.92-8.10) | 5.30(2.28-6.60) | 0.202 |
